# Mechanical and Electrical Noise in Sense Channel of MEMS Vibratory Gyroscopes

**DOI:** 10.3390/s17102306

**Published:** 2017-10-11

**Authors:** Xukai Ding, Jia Jia, Yang Gao, Hongsheng Li

**Affiliations:** 1 School of Instrument Science and Engineering, Southeast University, Nanjing 210096, China; dingxukai@126.com (X.D.); 230169207@seu.edu.cn (J.J.); 230139244@seu.edu.cn (Y.G.); 2 Key Laboratory of Micro-Inertial Instruments and Advanced Navigation Technology, Ministry of Education, Nanjing 210096, China

**Keywords:** angle random walk, force-rebalance, MEMS vibratory gyroscope, mode matching, noise analysis, noise equivalent rate, power spectral density, open-loop

## Abstract

This paper presents a theoretical analysis of mechanical and electrical noise in the sense channel of micro-electromechanical systems (MEMS) vibratory gyroscopes. Closed-form expressions for the power spectral density (PSD) of the noise equivalent rate (NER) of gyroscopes in the open-loop and the force-rebalance operations are derived by using an averaged PSD model and an equivalent transfer function. The obtained expressions are verified through numerical simulations, demonstrating close agreements between the analytic and the numerical models. Based on the derived expressions for the PSD of the NER, the impacts of the modal frequency split, quality factor, and the gain of the feedback forcer, as well as the gain of the signal conditioning circuit, on the gyroscope noise characteristics are theoretically analyzed. In addition, the angle random walk (ARW) and the standard deviation of the NER are also discussed through the PSD models. Finally, the effects of the loop closing, the mode matching, and the gain of the feedback forcer on the PSD of the NER were verified via a MEMS vibratory gyroscope with a tunable modal frequency split.

## 1. Introduction

Gyroscopes based on micro-electromechanical systems (MEMS) technologies have great advantages of small size, low power consumption, low cost, and batch fabrication over their traditional counterparts. MEMS vibratory gyroscopes have been widely adopted in various consumer electronics, as well as in industrial fields. However, the noise performance is one of the essential limitations of MEMS vibratory gyroscopes operating on high-precision occasions. Noise analysis of the gyroscope sense channel is of great importance to system designs. The impacts of different operations and system parameters on the noise performance can provide constructive guidance on determining a sufficient quality factor of the resonator, a reasonable modal frequency split, and adopting proper feedback forcers.

There have been several classic studies focusing on the noise analysis of MEMS vibratory gyroscopes. A comprehensive review of noise in MEMS devices is given in [1]. Noise in MEMS vibratory gyroscopes typically contains mechanical-thermal noise and electrical noise. The mechanical noise has a fundamental intrinsic mechanism, namely Brownian motion, which is due to dynamic unbalanced forces caused by random impacts of molecules on a structure. Leland investigated the mechanical-thermal noise in MEMS gyroscopes via the method of stochastic averaging [2]. The expressions for the impacts of the mechanical noise, including the angle random walk (ARW), the noise equivalent rate (NER), and the spectral density of the rate noise, are derived in [2]. Common electrical noise sources are summarized in [3], including electrical-thermal noise (Johnson noise), flicker noise, demodulation phase noise, and so on. In [4,5], Kim and M’Closkey presented a detailed noise spectral analysis of force-rebalance mode-matched vibratory gyroscopes. Expressions for the NER spectrum and the integrated angular rate variance are derived to discuss the impacts of modal frequency split, closed-loop bandwidth, and the noise sources at the sensor’s input and output.

In our previous work [6], the mechanical noise and the electrical noise in the force-rebalance operation was studied via numerical simulations. The challenge of the theoretical analysis in the force-rebalance operation is the nonlinear modulation–demodulation process in the feedback loop, making the linear analysis methods infeasible in this situation. In [2], the high- and low-frequency variables are isolated by using a coordinate transform. Then, stochastic averaging is applied to the transformed system to yield a system with slow variables. By this approach, an approximate linear system model, which is easy for the noise analysis, is obtained. However, the author only dealt with the mechanical-thermal noise. Since the electrical noise and the mechanical noise influence the system from different positions in the loop, the expressions derived in [2] cannot be directly applied to the electrical noise. Although the noise model in [5] includes both the mechanical noise and the electrical noise, the feedback architecture of the force-rebalance operation in the study is not a typical one. In a typical feedback scheme, as presented in [2], the output of the sense axis is firstly demodulated to extract the amplitude of Coriolis vibration, then fed to a controller, then modulated with a signal in-phase with the Coriolis force, and finally applied to the feedback forcer. In [5], the output signal of the sense axis is multiplied by a constant gain, and then directly fed back to the forcer, leaving in-phase and quadrature components indistinguishable. The only demodulation process in this scheme is at the output stage, where the Coriolis information is extracted. Considering there is no nonlinear process in the feedback loop, the closed-form expressions derived in [5] are inapplicable to the typical force-rebalance architecture.

In this paper, we obtain closed-form expressions for the power spectral density (PSD) of the mechanical and the electrical NER in a typical force-rebalance architecture by using an averaged PSD model and an equivalent transfer function of the modulated–demodulated sense axis. The PSD of the NER in gyroscope outputs can reflect the noise performance of the gyroscopes in great detail [7] and the relationship between the PSD and the Allan Variance is studied in [8]. In the open-loop noise model, the features of the noise after the demodulation process, which is nonlinear and will yield a non-stationary random signal, are investigated by the averaged PSD model. In the force-rebalance noise model, the noise sources are separated from the closed-loop, and the modulation–demodulation process in the closed-loop is linearized by the equivalent transfer function. By this approach, the closed-loop noise model can be transformed into an open-loop one and, hence, the analysis method used in the open-loop model can be applied. The analytic models are in close agreement with the numerical models, verifying the validity of the closed-form expressions. The analyses and simulations in this paper can reveal the noise propagation in the sense channel of MEMS vibratory gyroscopes and give important guidance on the system design.

MEMS vibratory gyroscopes in the force-rebalance operation, in contrast to those in the open-loop operation, exhibit better linearity, less sensitivity to environmental temperature variations, independence between bandwidth and mechanical sensitivity, and a tunable dynamic range [9,10,11]. However, the open-loop operation is less complicated and shows a sufficient level of performance in some applications. In consideration of the differences between the open-loop operation and the force-rebalance operation, in this paper, we will discuss the noise features in both of these two aspects. In addition, MEMS vibratory gyroscopes can be in mode-matched situations or mode-split situations depending on the frequency split between the drive axis and the sense axis. Mode-matched gyroscopes demonstrate higher mechanical sensitivities than mode-split gyroscopes [12,13,14,15]. Due to the difference of the mechanical responses between the mode-split and the mode-matched gyroscopes, their equivalent transfer functions are also different. Therefore, we will respectively analyze the mode-split and the mode-matched models.

Besides noise, bias drift is another important performance of gyroscopes. The bias drift is a result of the unstable behavior of the zero rate output of the gyroscopes due to environmental changes, especially to the change of temperature. The zero rate output of MEMS vibratory gyroscopes is studied in detail in [16]. Different operations and parameters of gyroscopes will change the zero rate output and, therefore, will certainly change the bias drift. However, considering the bias drift and the noise have different formation mechanisms, we will not discuss the effects of different operations and parameters on the bias drift in this paper.

This paper is organized as follows. Section 2 gives a general background of the sense channel model in MEMS vibratory gyroscopes, including the open-loop operation and the force-rebalance operation. The expressions for the frequency response of the gyroscope and the scale-factor are briefly introduced in both operations. The open-loop noise model is analyzed in Section 3. The noise propagation in the open-loop is demonstrated, and the closed-form expression for the PSD of the NER are derived. Section 4 presents the noise analysis of the force-rebalance model. The validity of the obtained closed-form expressions is verified through numerical simulations for both the mode-split and mode-matched gyroscopes. Through the obtained PSD models, the ARW and the standard deviation of the NER are discussed in Section 5. Some conclusions in Section 5 were verified via a MEMS vibratory gyroscope with a tunable frequency split, and the results are shown in Section 6. Finally, Section 7 concludes this paper with a summary.

## 2. Model of Sense Channel in MEMS Vibratory Gyroscopes

Typically a MEMS vibratory gyroscope can be modeled as a two-dimensional oscillator

(1) $$\begin{array}{c} m\ddot{x}-2m\eta \Omega \dot{y}+c_{x}\dot{x}+k_{x}x=F_{x}, \end{array}$$

(2) $$\begin{array}{c} m\ddot{y}+2m\eta \Omega \dot{x}+c_{y}\dot{y}+k_{y}y=F_{y}, \end{array}$$

where *m* is the mass of the oscillator; *x* and *y* are displacements along the two orthogonal dimensions, that is x- and y- axes; η is an angular gain factor, which is determined by the structural geometry of the gyroscope; Ω is the input angular rate; $c_{x}$ and $c_{y}$ represent the damping coefficients; $k_{x}$ and $k_{y}$ are stiffness; and $F_{x}$ and $F_{y}$ are external forces exerted on the x- and y- axes. $\omega_{x}=\sqrt{k_{x}/m}$ and $\omega_{y}=\sqrt{k_{y}/m}$ are natural frequencies of the x-axis and the y-axis, respectively. If $\omega_{x}=\omega_{y}$, gyroscopes are in the mode-matched situation; otherwise, gyroscopes are in the mode-split situation. The damping and the stiffness couplings are ignored in Equations (1) and (2) because these non-ideal components only introduce output bias and, accordingly, have no significant impacts on the gyroscope noise performance.

We refer to the x-axis of vibratory gyroscopes as the drive axis and the y-axis as the sense axis. The drive axis is excited into stable resonance by an external electrostatic force, $F_{x}$, whose frequency and amplitude are respectively regulated by a phase-locked loop (PLL) and an automatic gain control (AGC) loop. In the open-loop operation, there is no external force applied along the sense axis, which means $F_{y}=0$, and the sense axis is only driven by the Coriolis force. The vibration amplitude of the sense axis is proportional to the angular rate input and, therefore, can be used as a measure of the angular rate. In the force-rebalance operation, namely the closed-loop operation, an electrostatic force is applied to exactly balance the Coriolis force and to null the vibration of the sense axis. In this operation, the voltages applied to the feedback electrodes, which generate the electrostatic force, can be used as an estimation of the angular rate.

We assume the drive axis has a steady vibration under the control of the PLL and the AGC loop

(3) $$x\left(t\right)=A_{x}\sin\left(\omega_{d}t\right),$$

where $A_{x}$ is the vibration amplitude of the drive axis in meters, and $\omega_{d}$ is the drive frequency and always tracks $\omega_{x}$. Equation (3) indicates that the Coriolis force exerted on the sense axis is

(4) $$F_{c}=-2m\eta A_{x}\omega_{d}\Omega \cos\left(\omega_{d}t\right).$$

**Figure 1:**
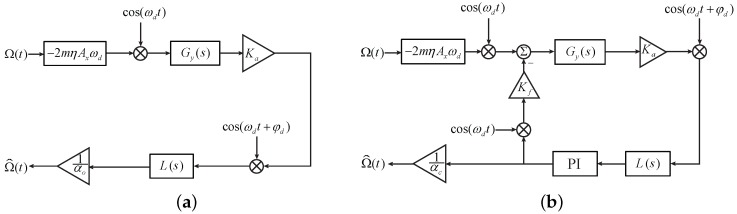
Block diagrams of the sense channel in MEMS vibratory gyroscopes. (**a**) The open-loop operation; (**b**) The force-rebalance operation.

We refer to the sense axis, together with the corresponding readout and control circuits, as the sense channel. Figure 1 demonstrates the general block diagrams of the sense channel in both the open-loop operation and the force-rebalance operation. Symbols in Figure 1 are explained as follows. $G_{y}\left(s\right)$ is the transfer function of the sense axis from applied forces to pickoff voltages

(5) $$G_{y}\left(s\right)=\frac{K_{xv}/m}{s^{2}+\omega_{y}s/Q_{y}+\omega_{y}^{2}},$$

where $K_{xv}$ is the pickoff circuit gain from displacement to voltage in V/m; and $Q_{y}=m\omega_{y}/c_{y}$ is the quality factor. $K_{a}$ is the gain of the signal conditioning circuit in V/V. L(s) is the transfer function of a low pass filter (LPF) with a cutoff frequency of $f_{l0}$. $\alpha_{o}$ and $\alpha_{c}$ are scale-factors of the open-loop operation and the force-rebalance operation, respectively. Module PI in Figure 1b is the feedback controller, which is commonly implemented by a proportional–integral controller. The gain of the feedback forcer, $K_{f}$, in N/V, is given by

(6) $$K_{f}=\frac{\partial C_{fb}}{\partial y}K_{DA}V_{D},$$

where $C_{fb}$ is the capacitance of the forcer electrodes, $K_{DA}$ is the gain of the buffer of the digital-to-analog converter, and $V_{D}$ is the bias direct current (DC) voltage applied to the forcer electrodes. $\varphi_{d}$ is a demodulation phase, whose optimal value is determined by

(7) $$\varphi_{od}=-\tan^{-1}\frac{\omega_{y}\omega_{d}/Q_{y}}{\omega_{y}^{2}-\omega_{d}^{2}}.$$

In practice, $\varphi_{d}$ will be set as 0 for mode-split gyroscopes and −π/2 for mode-matched gyroscopes.

**Figure 2:**
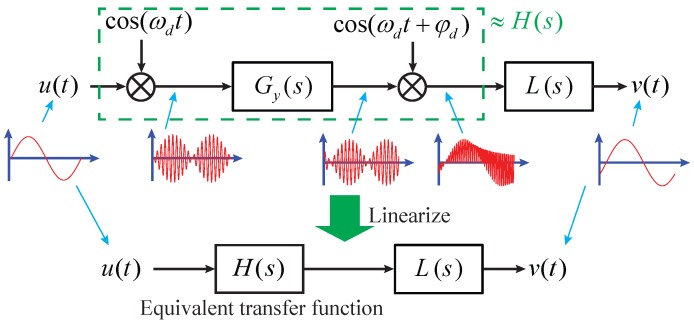
Equivalent transfer function of the modulation–demodulation process in the gyroscope sense channels.

Considering the modulation–demodulation process shown in Figure 2, the equivalent transfer function can be expressed as [17]

(8) $$H\left(s\right)\approx \frac{K_{xv}}{m}\frac{\left(\omega_{y}^{2}-\omega_{d}^{2}\right)\cos\varphi_{d}/2-\left(\omega_{d}s+\omega_{y}\omega_{d}/2Q_{y}\right)\sin\varphi_{d}}{{\left(\omega_{d}+\omega_{y}\right)}^{2}\left[s^{2}+\omega_{y}s/Q_{y}+{\left(\omega_{y}-\omega_{d}\right)}^{2}+\omega_{y}\omega_{d}/4Q_{y}^{2}\right]},$$

where the approximation is taken when $Q_{y}>>1/2$ and $\omega_{y,d}>>2\pi f_{l0}$.

By applying this linearization method to Figure 1a, we obtain the equivalent transfer function of the open-loop sense channel as

(9) $$\frac{\hat{\Omega }\left(s\right)}{\Omega (s)}=-\frac{2\eta mA_{x}\omega_{d}}{\alpha_{o}}K_{a}H\left(s\right)L\left(s\right),$$

and the open-loop scale-factor as

(10) $$\begin{array}{cc} \alpha_{o} & =-2\eta mA_{x}\omega_{d}K_{a}{H\left(s\right)L\left(s\right)|}_{s=0}\\ & =-\frac{2\eta A_{x}\omega_{d}K_{a}K_{xv}}{{\left(\omega_{d}+\omega_{y}\right)}^{2}}\frac{\left(\omega_{y}^{2}-\omega_{d}^{2}\right)\cos\varphi_{d}/2-\omega_{d}\omega_{y}\sin\varphi_{d}/2Q_{y}}{\Delta \omega^{2}+\omega_{y}\omega_{d}/4Q_{y}^{2}}, \end{array}$$

where $\Delta \omega ≜\omega_{y}-\omega_{d}$.

**Figure 3:**
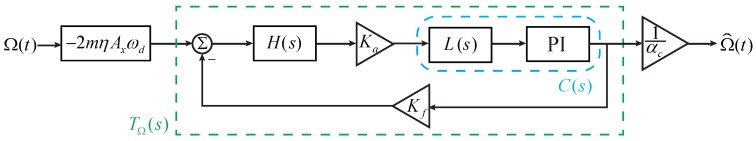
The equivalent block diagram of the force-rebalance sense channel.

Similarly, the block diagram of the force-rebalance operation in Figure 1b can be equivalent to the one shown in Figure 3. For convenience, we denote

(11) $$C\left(s\right)=L\left(s\right)\left(K_{p}+\frac{K_{i}}{s}\right),$$

where $K_{p}$ and $K_{i}$ are parameters of the PI controller. Considering

(12) $$T_{\Omega }\left(s\right)=\frac{K_{a}H\left(s\right)C\left(s\right)}{1+K_{a}K_{f}H\left(s\right)C\left(s\right)},$$

the overall transfer function of the force-rebalance sense channel is

(13) $$\frac{\hat{\Omega }\left(s\right)}{\Omega (s)}=-\frac{2\eta mA_{x}\omega_{d}}{\alpha_{c}}T_{\Omega }\left(s\right),$$

and the force-rebalance scale-factor is

(14) $$\alpha_{c}=-\frac{2\eta mA_{x}\omega_{d}}{K_{f}}.$$

Equations (9) and (13) respectively give the gyroscope frequency responses in the open-loop operation and the force-rebalance operation to the applied angular rate.

## 3. Open-Loop Noise Model

**Figure 4:**
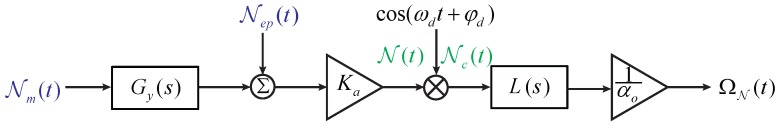
The block diagram of the open-loop noise model.

To evaluate the noise characteristics in the open-loop sense channel, we ignore the angular rate input and introduce the mechanical and the electrical noise, as depicted in Figure 4. $N_{m}\left(t\right)$ is mechanical-thermal noise in Newton. $N_{ep}\left(t\right)$ is the electrical noise brought in by the pickoff and the signal conditioning circuits, including the flicker noise and the Johnson noise. $\Omega_{N}\left(t\right)$ is the measured NER in the gyroscope output.

Several assumptions are made in this model for the noise analysis.

(1) The vibration of the drive axis under the electrostatic force excitation is sufficiently greater than the mechanical-thermal disturbance. Therefore, the NER coupling from the amplitude noise of the drive axis through Coriolis effect is insignificant and can be ignored.
(2) Considering the drift of $\omega_{x}$ is extremely slow, the control parameters of the PLL in the drive axis can be set to achieve a very low phase noise for the frequency tracking. Consequently, the phase noise in the demodulation process is neglected in this paper.
(3) Generally, there are multiple electrical noise sources in the system. However, in linear systems, multi stage noise sources can be equivalently converted into the one at the first stage [18]. In analog systems, the noise sources after the demodulation, typically introduced by the LPF, are trivial and thus can be neglected. In digital systems, no additional electrical noise will be presented after the analog-to-digital conversion. Taking these facts into account, we only model one electrical noise source at the pickoff node.
(4) In addition, digitally-based noise, namely spurious, which is usually introduced by clocks and power supplies, is also ignored in this paper.

The PSD of the mechanical noise and the electrical noise can be respectively described by

(15) $$\begin{array}{c} S_{Nm}\left(f\right)=2k_{B}Tc_{y},\;-\infty <f<+\infty , \end{array}$$

(16) $$\begin{array}{c} S_{Nep}\left(f\right)=2k_{B}TR_{p}+k_{1}/\left|f\right|,\;-\infty <f<+\infty , \end{array}$$

where *f* is the frequency in Hertz, $k_{B}$ is the Boltzmann constant, *T* is the temperature in Kelvin, $R_{p}$ represents an equivalent resistance for the Johnson noise, and $k_{1}$ is a constant for the flicker noise. Double-side spectral models are adopted in Equations (15) and (16) for the convenience of theoretical analysis. It should be noted that the equivalent resistance $R_{p}$ is not the resistance of a real resistor but a variable representing the Johnson noise equivalently. The noise performance of the pickoff circuit can be evaluated by the ratio of $\sqrt{S_{Nep}\left(f\right)}/K_{xv}$, which is always preferred as low as possible.

The PSD of N(t) in Figure 4 can be readily obtained as

(17) $$S_{N}\left(f\right)=K_{a}^{2}{\left|G_{y}\left(f\right)\right|}^{2}S_{Nm}\left(f\right)+K_{a}^{2}S_{Nep}\left(f\right),$$

where

(18) $$\begin{array}{cc} |G_{y}{\left(f\right)|}^{2} & =|G_{y}{\left(s\right)|}_{s=j2\pi f}{|}^{2}\\ & =\frac{K_{xv}^{2}/m^{2}}{{\left[\omega_{y}^{2}-{\left(2\pi f\right)}^{2}\right]}^{2}+{\left(2\pi f\omega_{y}/Q_{y}\right)}^{2}}. \end{array}$$

To scrutinize the PSD of the modulated noise $N_{c}\left(t\right)=N\left(t\right)\cdot \cos\left(\omega_{d}t+\varphi_{d}\right)$, we examine its autocorrelation function (ACF) via

(19) $$\begin{array}{cc} R_{N_{c}}\left(\tau ,t\right) & =E[N_{c}\left(t\right)N_{c}\left(t+\tau \right)]\\ & =R_{N}\left(\tau \right)\frac{1}{2}\left[\cos\left(\omega_{d}\tau \right)+\cos\left(2\omega_{d}t+\omega_{d}\tau +2\varphi_{d}\right)\right], \end{array}$$

where E[·] denotes the expectation operator, and $R_{N}\left(\tau \right)$ represents the ACF of N(t). Equation (19) states that $N_{c}\left(t\right)$ is not wide-sense stationary due to its time dependence. However, because $R_{N_{c}}\left(\tau ,t\right)$ is a fast-changing variable with respect to time, we can find an averaged ACF of $N_{c}\left(t\right)$ as

(20) $${\tilde{R}}_{N_{c}}\left(\tau \right)=\frac{1}{2}\cos\left(\omega_{d}\tau \right)R_{N}\left(\tau \right)$$

over a period of $\pi /\omega_{d}$. Accordingly, applying Fourier transform to Equation (20) and denoting $f_{d}≜\omega_{d}/2\pi$ give the averaged PSD of $N_{c}\left(t\right)$

(21) $$\begin{array}{cc} {\tilde{S}}_{N_{c}}\left(f\right) & =\frac{1}{4}\left[S_{N}\left(f+f_{d}\right)+S_{N}\left(f-f_{d}\right)\right]\\ & =\frac{K_{a}^{2}}{4}2k_{B}T\left[c_{y}|G_{y}\left(f+f_{d}\right){|}^{2}+c_{y}{\left|G_{y}\left(f-f_{d}\right)\right|}^{2}+2R_{p}\right]\\ & \;+\frac{K_{a}^{2}}{4}\left(\frac{k_{1}}{|f+f_{d}|}+\frac{k_{1}}{|f-f_{d}|}\right), \end{array}$$

where

(22) $$\begin{array}{cc} |G_{y}\left(f+f_{d}\right){|}^{2} & \approx \frac{K_{xv}^{2}/m^{2}}{4\omega_{y}^{2}{\left(\Delta \omega -2\pi f\right)}^{2}+\omega_{y}^{4}/Q_{y}^{2}}, \end{array}$$

(23) $$\begin{array}{cc} |G_{y}\left(f-f_{d}\right){|}^{2} & \approx \frac{K_{xv}^{2}/m^{2}}{4\omega_{y}^{2}{\left(\Delta \omega +2\pi f\right)}^{2}+\omega_{y}^{4}/Q_{y}^{2}}. \end{array}$$

The approximations in Equations (22) and (23) hold under the conditions that $\Delta \omega <<\omega_{y}$ and $2\pi f<<\omega_{y}$.

**Figure 5:**
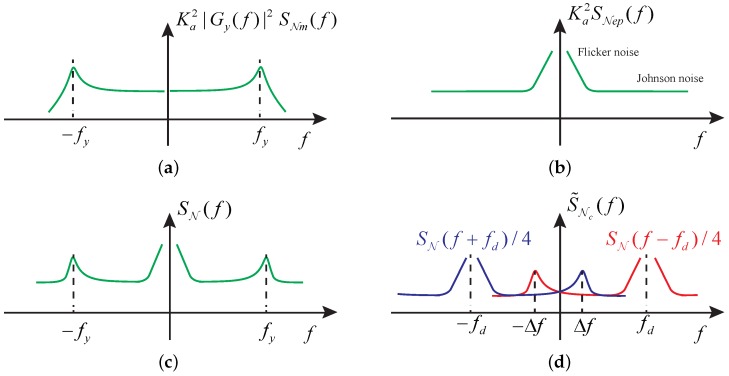
Noise propagation in the open-loop sense channel. (**a**) Noise power spectral density (PSD) in N(t) attributable to the mechanical noise; (**b**) Noise PSD in N(t) attributable to the electrical noise; (**c**) Noise synthesis of N(t); (**d**) Noise PSD after the modulation. Δf=Δω/2π.

Figure 5 visualizes the noise PSD described by Equations (15)–(17) and (21). For the mechanical noise in N(t), the PSD at $f_{y}=\omega_{y}/2\pi$ is larger than those in low frequencies by a factor of $Q_{y}^{2}$, predicted by Equation (18). The flicker noise is pronounced in low frequencies, and the Johnson noise prevails in high frequencies. The crossover frequency, at which the PSD of the flicker noise equals that of the Johnson noise, is generally from several Hertz to tens of Hertz. As depicted in Figure 5c, the PSD feature of N(t) can be utilized to identify the mechanical noise and the electrical noise for situations where the Johnson noise PSD is below the peak of the mechanical noise PSD. Once the peak magnitude of the mechanical noise PSD at the resonant frequency is obtained, the overall PSD of the mechanical noise can be determined via Equation (18). An example of this procedure will be demonstrated in Section 6.

Considering the low-pass feature of L(s), the modulated flicker noise can be sufficiently suppressed in the gyroscope output, yielding the PSD of the NER as

(24) $$\begin{array}{cc} {\tilde{S}}_{\Omega_{N}}\left(f\right) & ={\tilde{S}}_{N_{c}}\left(f\right)\frac{{\left|L\left(f\right)\right|}^{2}}{\alpha_{o}^{2}}\\ & \approx \frac{{\left|L\left(f\right)\right|}^{2}}{4\alpha_{o}^{2}}K_{a}^{2}2k_{B}T\left[2R_{p}+c_{y}|G_{y}\left(f+f_{d}\right){|}^{2}+c_{y}{\left|G_{y}\left(f-f_{d}\right)\right|}^{2}\right], \end{array}$$

where ${L\left(f\right)=L\left(s\right)|}_{s=j2\pi f}$.

For mode-split gyroscopes, provided that $\Delta \omega >>\omega_{y}/2Q_{y}$, the open-loop scale-factor (Equation (10)) can be simplified as

(25) $$\alpha_{o}\approx -\frac{\eta A_{x}K_{a}K_{xv}}{2\Delta \omega },$$

where the modulation phase $\varphi_{d}$ is taken as 0. By combining Equations (24) and (25), we obtain the expression of the PSD of the NER for open-loop mode-split gyroscopes as

(26) $${\tilde{S}}_{\Omega_{N}}\left(f\right)={\left|L\left(f\right)\right|}^{2}\frac{2k_{B}T\Delta \omega^{2}}{\eta^{2}A_{x}^{2}}\left\{\frac{2R_{p}}{K_{xv}^{2}}+[\frac{1/m\omega_{y}Q_{y}}{4{\left(\Delta \omega -2\pi f\right)}^{2}+\omega_{y}^{2}/Q_{y}^{2}}+\frac{1/m\omega_{y}Q_{y}}{4{\left(\Delta \omega +2\pi f\right)}^{2}+\omega_{y}^{2}/Q_{y}^{2}}]\right\}.$$

**Figure 6:**
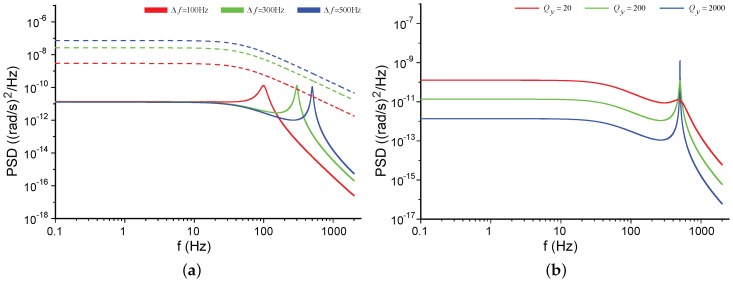
The PSD of the noise equivalent rate (NER) for different parameters in open-loop mode-split gyroscopes. Solid lines for the mechanical noise. Dashed lines for the electrical noise. (**a**) Comparisons between different Δf; (**b**) Comparisons between different $Q_{y}$.

Figure 6 shows the influences of the modal frequency split Δf and the quality factor $Q_{y}$ on the NER. The negative frequency parts of the plots are not shown here for the even property of ${\tilde{S}}_{\Omega_{N}}\left(f\right)$. The default parameter values used in this paper are listed in Table 1 unless some of them are re-specified in the context or figures. In Figure 6a, the PSD of the mechanical NER is not notably affected by Δf in low frequencies, but its peak shifts with Δf; the PSD of the electrical NER increases as Δf grows. The PSD of the mechanical NER is inversely proportional to $Q_{y}$ at low frequencies but proportional to $Q_{y}$ at the frequency of Δf, Figure 6b. The PSD of the electrical NER will not be affected by $Q_{y}$, revealed by Equation (26), and hence their relationship is not presented in Figure 6b for the sake of clarity.

For mode-matched gyroscopes, Δω=0, hence $\varphi_{d}$ should be set as −π/2, and the open-loop scale-factor (Equation (10)) can be simplified as

(27) $$\alpha_{o}\approx -\frac{\eta A_{x}K_{a}K_{xv}Q_{y}}{\omega_{y}}.$$

Equations (24) and (27) give the PSD of the NER for open-loop mode-matched gyroscopes

(28) $${\tilde{S}}_{\Omega_{N}}\left(f\right)={\left|L\left(f\right)\right|}^{2}\frac{k_{B}T\omega_{y}^{2}}{\eta^{2}A_{x}^{2}Q_{y}^{2}}\left[\frac{R_{p}}{K_{xv}^{2}}+\frac{1}{m\omega_{y}Q_{y}\left(16\pi^{2}f^{2}+\omega_{y}^{2}/Q_{y}^{2}\right)}\right].$$

**Figure 7:**
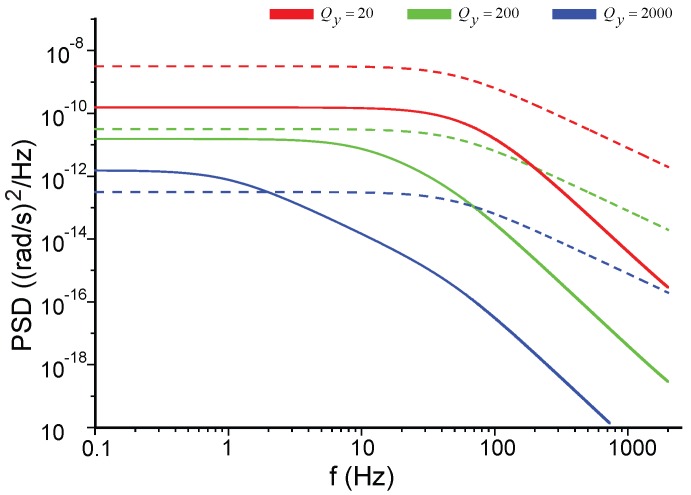
The PSD of the NER for different $Q_{y}$ in open-loop mode-matched gyroscopes. Solid lines for the mechanical noise. Dashed lines for the electrical noise.

The relationships between the NER and $Q_{y}$ are shown in Figure 7, which clearly demonstrates that the boost of $Q_{y}$ can reduce both the mechanical NER and the electrical NER. High $Q_{y}$ improves the electrical NER in open-loop mode-matched gyroscopes because the sensitivity of these gyroscopes is directly proportional to $Q_{y}$, Equation (27). However, on account of the poor environmental robustness of $Q_{y}$, mode-matched gyroscopes seldom work in the open-loop operation.

## 4. Force-Rebalance Noise Model

**Figure 8:**
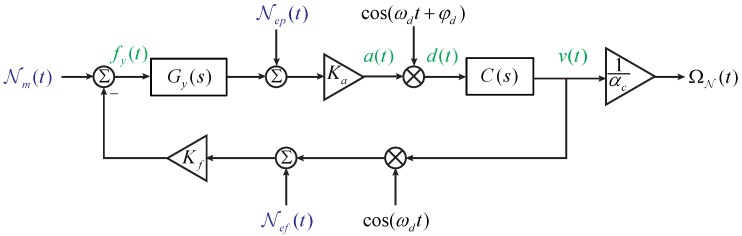
The block diagram of the force-rebalance noise model.

In the force-rebalance sense channel, besides the noise sources introduced in the open-loop, we model another electrical noise source $N_{ef}\left(t\right)$ at the feedback node, as demonstrated in Figure 8. The reason is that $N_{ef}\left(t\right)$ is separated from $N_{ep}\left(t\right)$ by the modulation–demodulation process in the loop and, as a result, cannot be equivalent to $N_{ep}\left(t\right)$. The electrical noise source at the output stage is still ignored for the same reason discussed in Section 3.

**Figure 9:**
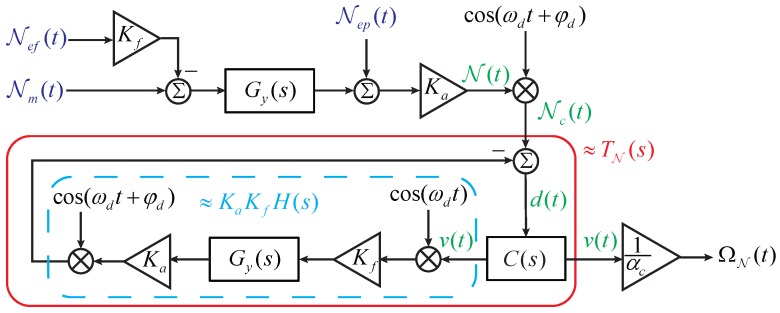
The equivalent noise model of the force-rebalance sense channel.

Different from the open-loop operation, noise sources of the force-rebalance operation are in the closed-loop, making the noise propagate circularly. The basic idea is to isolate the noise sources from the closed-loop to make the analysis straightforward. Consider the system dynamics of the model demonstrated in Figure 8

(29) $$\begin{array}{c} f_{y}\left(t\right)=-K_{f}\left[v\left(t\right)\cos\left(\omega_{d}t\right)+N_{ef}\left(t\right)\right]+N_{m}\left(t\right), \end{array}$$

(30) $$\begin{array}{c} a\left(t\right)=K_{a}\left[f_{y}\left(t\right)*g_{y}\left(t\right)+N_{ep}\left(t\right)\right], \end{array}$$

(31) $$\begin{array}{c} d\left(t\right)=a\left(t\right)\cos\left(\omega_{d}t+\varphi_{d}\right), \end{array}$$

(32) $$\begin{array}{c} v(t)=d(t)*c(t), \end{array}$$

(33) $$\begin{array}{c} \Omega_{N}\left(t\right)=v\left(t\right)/\alpha_{c}, \end{array}$$

where ∗ denotes the convolve operation, and $g_{y}\left(t\right)$ and c(t) are inverse Laplace transforms of $G_{y}\left(s\right)$ and C(s), respectively. Through simple algebraic manipulations of above equations, we get

(34) $$\begin{array}{cc} d(t)= & K_{a}N_{m}\left(t\right)*g_{y}\left(t\right)\cos\left(\omega_{d}t+\varphi_{d}\right)+K_{a}N_{ep}\left(t\right)\cos\left(\omega_{d}t+\varphi_{d}\right)\\ & -K_{a}K_{f}N_{ef}\left(t\right)*g_{y}\left(t\right)\cos\left(\omega_{d}t+\varphi_{d}\right)-K_{a}K_{f}d\left(t\right)*c\left(t\right)\cos\omega_{d}t*g_{y}\left(t\right)\cos\left(\omega_{d}t+\varphi_{d}\right), \end{array}$$

which can be translated into the model presented in Figure 9. Via this approach, the noise sources are all separated from the closed-loop.

By applying the linearization method presented in Figure 2 to the model presented in Figure 9, we can easily obtain

(35) $$T_{N}\left(s\right)=\frac{C(s)}{1+K_{a}K_{f}C\left(s\right)H\left(s\right)}.$$

In Figure 9, the noise propagation in the force-rebalance operation is close to that in the open-loop operation

(36) $$\begin{array}{c} S_{N}\left(f\right)=K_{a}^{2}{\left|G_{y}\left(f\right)\right|}^{2}\left[S_{Nm}\left(f\right)+K_{f}^{2}S_{Nef}\left(f\right)\right]+K_{a}^{2}S_{Nep}\left(f\right), \end{array}$$

(37) $$\begin{array}{c} {\tilde{S}}_{N_{c}}\left(f\right)=\frac{1}{4}\left[S_{N}\left(f+f_{d}\right)+S_{N}\left(f-f_{d}\right)\right], \end{array}$$

(38) $$\begin{array}{c} {\tilde{S}}_{\Omega_{N}}\left(f\right)={\tilde{S}}_{N_{c}}\left(f\right){\left|T_{N}\left(f\right)/\alpha_{c}\right|}^{2}, \end{array}$$

where $S_{Nef}\left(f\right)$ is the PSD of $N_{ef}\left(t\right)$

(39) $$S_{Nef}\left(f\right)=2k_{B}TR_{f}+k_{2}/\left|f\right|,$$

and

(40) $$T_{N}\left(f\right)=T_{N}{\left(s\right)|}_{s=j2\pi f}.$$

Combining Equations (15), (16) and (36)–(39) yields

(41) $${\tilde{S}}_{\Omega_{N}}\left(f\right)\approx \frac{|T_{N}{\left(f\right)|}^{2}}{4\alpha_{c}^{2}}K_{a}^{2}2k_{B}T\left\{\left(c_{y}+K_{f}^{2}R_{f}\right)\left[|G_{y}\left(f+f_{d}\right){|}^{2}+{\left|G_{y}\left(f-f_{d}\right)\right|}^{2}\right]+2R_{p}\right\},$$

where the modulated flicker noise is ignored in consideration of the low-pass feature of $T_{N}\left(s\right)$.

It should be noted that thanks to the modulation and the followed filtering process, the flicker noise in the loop, either open-loop or closed-loop, does not contribute to the NER in the output of gyroscopes. In practice, the NER that resulted from the flicker noise is believed to be introduced by the electrical noise source at the output stage.

Equations (12) and (35) share a common denominator, suggesting that the PSD of the NER and the frequency response of the gyroscope have magnitude peaks at the same frequencies.

In mode-split cases, Equation (8) can be further simplified to

(42) $$H\left(s\right)\approx \frac{K_{xv}}{4m\omega_{y}}\frac{\Delta \omega }{s^{2}+\omega_{y}s/Q_{y}+\Delta \omega^{2}}.$$

**Figure 10:**
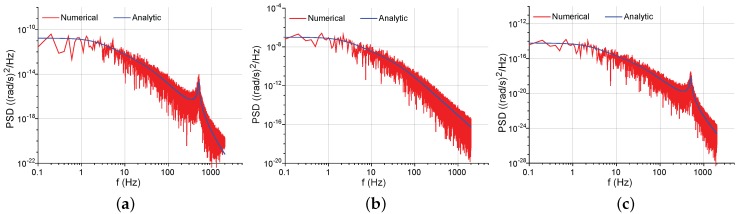
Numerically simulated and analytic PSD of the NER in the mode-split force-rebalance sense channel. (**a**) The mechanical NER; (**b**) The pickoff electrical NER; (**c**) The feedback electrical NER.

In Figure 10, the numerically simulated PSD curves are acquired via the noise model in Figure 8, while the analytic PSD curves are obtained through the closed-form Equation (41), along with Equations (14), (22), (23), (35) and (42). The consistency between the numerical and analytic curves manifests that the closed-form expressions can effectively describe the force-rebalance noise model in Figure 8. The mechanical NER and the feedback electrical NER are both determined by $T_{N}\left(s\right)$ and $G_{y}\left(s\right)$, leading to similar PSD shapes in Figure 10a,c. By contrast, the pickoff electrical NER is determined by $T_{N}\left(s\right)$ only.

**Figure 11:**
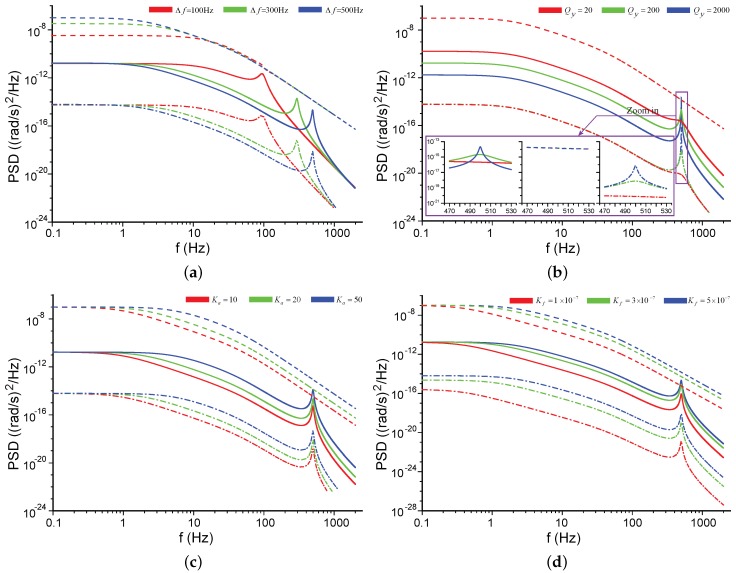
The PSD of the NER for different parameters in force-rebalance mode-split gyroscopes. Solid lines for the mechanical NER. Dashed lines for the pickoff electrical NER. Dot-dashed lines for the feedback electrical NER. (**a**) Comparisons between different modal frequency splits; (**b**) Comparisons between different $Q_{y}$; (**c**) Comparisons between different gains of the signal conditioning circuit; (**d**) Comparisons between different gains of the feedback forcer.

Figure 11 illustrates the impacts of Δf, $Q_{y}$, $K_{a}$, and $K_{f}$ on the NER. Similar to the relationships exhibited in Figure 6, in low frequencies, the PSD of the mechanical NER is not influenced by the modal frequency split, and the PSD of the pickoff electrical NER increases as the split increases, Figure 11a. The PSD of the mechanical NER is inversely proportional to $Q_{y}$ while the PSD of the pickoff electrical NER is not affected by $Q_{y}$, Figure 11b. The PSD shapes of the feedback electrical NER and those of the mechanical NER are very close except that (1) the PSD of the feedback electrical NER does not depend on $Q_{y}$ in low frequencies whereas that of the mechanical NER does, Figure 11b, and (2) the PSD of the feedback electrical NER will be changed by $K_{f}$ whereas that of the mechanical NER is relatively irrelevant to $K_{f}$ in low frequencies, Figure 11d. Here, we pay more attention to the PSD values in low frequencies because those values are directly related to the ARW of gyroscopes, which we will discuss in Section 5.

In mode-matched cases, Equation (8) will degenerate to

(43) $$H\left(s\right)\approx \frac{K_{xv}}{4m\omega_{y}}\frac{1}{s+\omega_{y}/2Q_{y}}.$$

**Figure 12:**
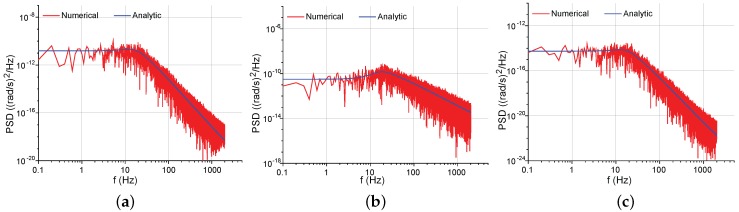
Numerically simulated and analytic PSD of the NER in the mode-matched force-rebalance sense channel. (**a**) The mechanical NER; (**b**) The pickoff electrical NER; (**c**) The feedback electrical NER.

Equations (14), (22), (23), (35), (41) and (43) give the closed-form expressions of the PSD of the NER in force-rebalance mode-matched gyroscopes. Figure 12 presents the corresponding analytic and numerically simulated PSD curves. In this operation, the controller parameters are set as $K_{p}=1$ and $K_{i}=120$. The analytic curves are in close agreement with the numerical ones, indicating the validity of the equivalent noise model.

**Figure 13:**
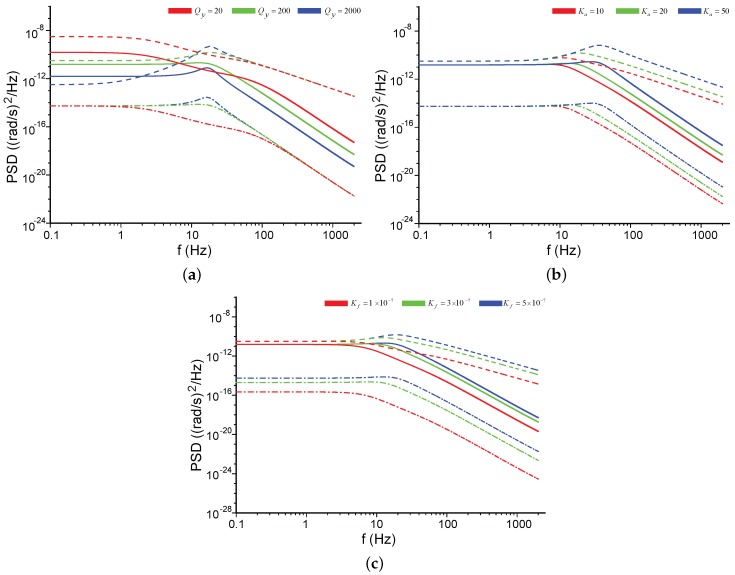
The PSD of the NER for different parameters in force-rebalance mode-matched gyroscopes. Solid lines for the mechanical NER. Dashed lines for the pickoff electrical NER. Dot-dashed lines for the feedback electrical NER. (**a**) Comparisons between different $Q_{y}$; (**b**) Comparisons between different gains of the signal conditioning circuit; (**c**) Comparisons between different gains of the feedback forcer.

Comparisons of the PSD of the NER between different $Q_{y}$, $K_{a}$, and $K_{f}$ are demonstrated in Figure 13. For mode-matched gyroscopes working in the force-rebalance operation, the enhancement of $Q_{y}$ can significantly improve the mechanical and the pickoff electrical noise. Similar to mode-split gyroscopes in the force-rebalance operation, in low frequencies, $K_{f}$ only affects the PSD of the feedback electrical NER. From Figure 11c and Figure 13b, the gain of the signal conditioning circuit will not influence the PSD of the NER in low frequencies for both the mode-split and the mode-matched cases.

## 5. Angle Random Walk and Standard Deviation of Noise Equivalent Rate

The random noise of gyroscopes is typically characterized by ARW, which can be derived from the Allan Variance of the gyroscope output data, at an integration time of 1 s. The ARW is related to the PSD of the NER via [2,8]

(44) $$\Omega_{ARW}\approx \sqrt{{\tilde{S}}_{\Omega_{N}}\left(0\right)}.$$

According to the PSD of the NER discussed in Section 3 and Section 4, the values of ${\tilde{S}}_{\Omega_{N}}\left(0\right)$ for gyroscopes in different operations are summarized in Table 2.

Table 2 reveals several interesting conclusions.

(1) The increase in $A_{x}$ can improve the ARW attributable to all noise components, which suggests an effective approach to improve the noise performance.
(2) The boost of $Q_{y}$ can reduce the ARW resulted from the mechanical noise in all of the operation cases but only decreases the ARW, to which the pickoff electrical noise contributes, in mode-matched cases.
(3) Mode matching will not influence the ARW from the mechanical noise but will affect the ARW from the pickoff electrical noise by a factor of $\Delta \omega /(\omega_{y}/2Q_{y})$.
(4) The enhancement of the pickoff circuit, namely a decrease in the ratio of $\sqrt{S_{Nep}\left(f\right)}/K_{xv}$, can reduce the ARW introduced by the pickoff electrical noise but does not affect the mechanically induced ARW.
(5) The gain of the feedback forcer only affects the ARW from the feedback electrical noise. In other words, in a well-designed system, if the feedback electrical noise is insignificant, the design value of $K_{f}$ is non-essential for the noise consideration.
(6) For a given gyroscope, loop closing of the sense channel, namely changing the sense channel from open-loop to force-rebalance, will not change the ARW from the mechanical noise and the pickoff electrical noise, neither in the mode-split case nor the mode-matched case.

In some applications, the standard deviation of the gyroscope output, $\sigma_{\Omega }$, in a specified frequency band, is utilized to specify random drift [7]. The standard deviation of the NER,

(45) $$\sigma_{\Omega_{N}}=\sqrt{\int_{-B}^{B}{\tilde{S}}_{\Omega_{N}}\left(f\right)df},$$

gives the lower limit of $\sigma_{\Omega }$, assuming that the output bias does not drift. *B* in Equation (45) is the frequency band of interest and is generally comparable to the gyroscope bandwidth. Signals with frequencies far beyond the gyroscope bandwidth do not contain effective rate information and, consequently, can be smoothed or averaged.

Based on Equation (45), the calculation of the standard deviation of the NER is very straightforward in open-loop sense channels. In mode-split cases,

(46) $$\sigma_{\Omega_{N}}^{2}=\frac{2k_{B}T\Delta \omega^{2}}{\eta^{2}A_{x}^{2}}\left\{\frac{4R_{p}B}{K_{xv}^{2}}+\frac{\tan^{-1}\left[2\left(\Delta \omega +2\pi B\right)Q_{y}/\omega_{y}\right]-\tan^{-1}\left[2\left(\Delta \omega -2\pi B\right)Q_{y}/\omega_{y}\right]}{2\pi m\omega_{y}^{2}}\right\},$$

and in mode-matched cases,

(47) $$\sigma_{\Omega_{N}}^{2}=\frac{2k_{B}T\omega_{y}^{2}}{\eta^{2}A_{x}^{2}4Q_{y}^{2}}\left[\frac{4R_{p}B}{K_{xv}^{2}}+\frac{\tan^{-1}\left(4\pi BQ_{y}/\omega_{y}\right)}{\pi m\omega_{y}^{2}}\right],$$

where the LPF is assumed as

(48) $$\left|L\left(f\right)\right|=\left\{\begin{array}{cc} 1, & \mathrm{if}|f|<B\\ 0, & \mathrm{otherwise}. \end{array}\right.$$

Considering the complexity of the expressions in force-rebalance channels, the corresponding $\sigma_{\Omega_{N}}$ can be discretely estimated via

(49) $$\sigma_{\Omega_{N}}^{2}\approx 2f_{res}\sum_{n=1}^{B/f_{res}}{\tilde{S}}_{\Omega_{N}}\left(n\cdot f_{res}\right),$$

where $f_{res}$ is the frequency resolution.

## 6. Experiments and Results

**Figure 14:**
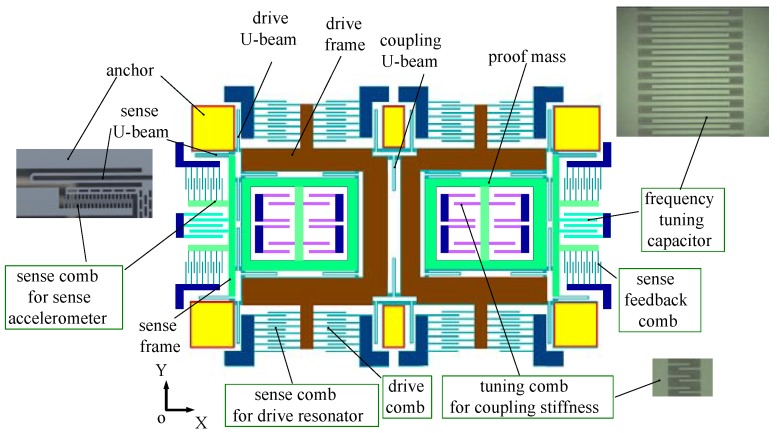
The silicon micro-gyroscope evaluated in the experiments [15].

The noise characteristics of MEMS vibratory gyroscopes were evaluated by a silicon tuning folk micro-gyroscope with a tunable modal frequency split, previously reported in [15]. Due to the negative stiffness effect, the resonant frequency of the sense axis can be lowered by DC voltages over the frequency tuning electrodes shown in Figure 14, which presents the schematic of the gyroscope design. For the gyroscope under the test, the natural frequency of the drive axis was 3563 Hz with $Q_{x}=2284$, and the natural frequency of the sense axis was 3629 Hz with $Q_{y}=187$, indicating a modal frequency split of 66 Hz. The modal frequency split could be tuned to 0 with a DC voltage of 8.2 V. The control system of the gyroscope was implemented by a field-programmable gate array (FPGA). 

**Figure 15:**
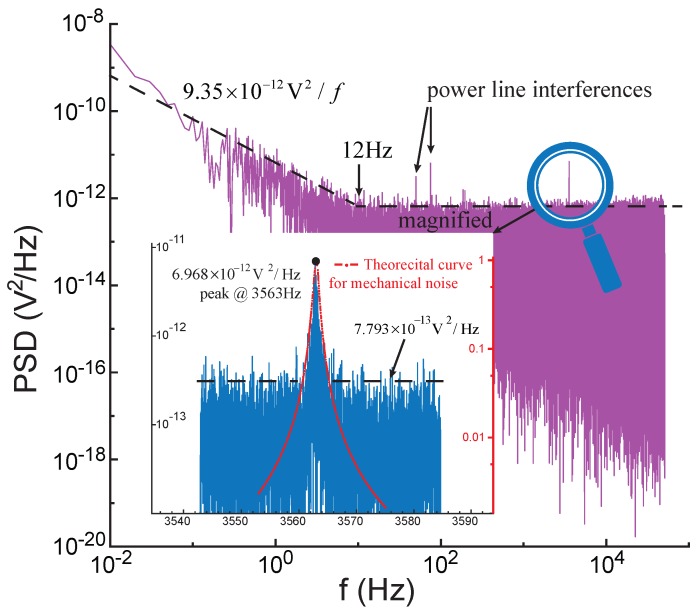
The PSD of the noise in the pickoff circuit. The theoretical curve in the insert was obtained via Equation (18).

Firstly, the method to identify the mechanical noise and the electrical noise mentioned in Section 3 was verified through the primary resonator (drive axis) of the gyroscope. The output of the pickoff circuit was recorded by a dynamic signal analyzer at a sampling frequency of 102.4 kHz for 100 s when the resonator was kept stationary. The resultant frequency resolution was 0.01 Hz. Figure 15 demonstrates the PSD of the acquired signal, which is similar to the right half PSD shown in Figure 5c. The crossover frequency of the electrical noise was measured as 12 Hz, and the PSD of the electrical-thermal noise was $7.793\times 10^{-13}\;V^{2}/\mathrm{Hz}$. A magnitude peak at the natural frequency of the resonator could be clearly observed, suggesting the mechanical noise PSD at the natural frequency was $6.968\times 10^{-12}\;V^{2}/\mathrm{Hz}$. According to Equation (18), the PSD of the mechanical noise in low frequencies could be estimated as $1.336\times 10^{-18}\;V^{2}/\mathrm{Hz}$, which was much smaller than that of the electrical-thermal noise. By this approach, we can separate the mechanical noise from the very noisy electrical noise, as long as the magnitude peak of the mechanical noise PSD can be notably observed.

**Figure 16:**
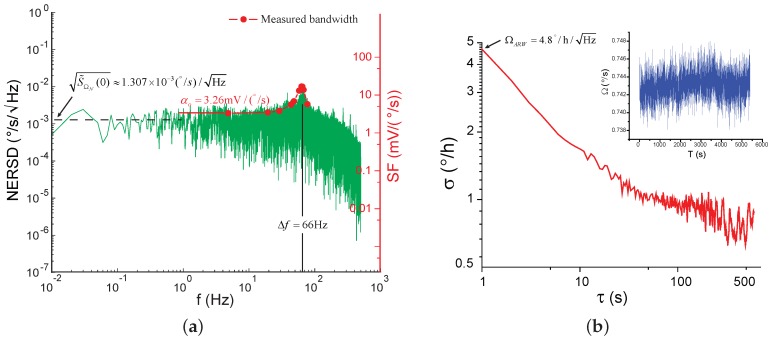
The NER spectral density of the gyroscope in the open-loop mode-split case. (**a**) The NER spectral density and the measured frequency response of the gyroscope; (**b**) The Allan Variance of the gyroscope output.(Insert: the time series of the recorded data.)

Then, the gyroscope was configured to operate in the open-loop mode-split case. Figure 16a shows the corresponding spectral density of the NER, along with the measured frequency response of the gyroscope. The output rate of the gyroscope was 1 kHz and the recording time was 100 s. The frequency response of the gyroscope was measured by an angular vibration table from 5 Hz to 80 Hz. The spectral density of the NER and the gyroscope frequency response had magnitude peaks at the same frequency, as explained in Section 4. In Figure 16a, $\sqrt{{\tilde{S}}_{\Omega_{N}}\left(0\right)}\approx 1.307\times 10^{-3}{(}^{\circ }/s)/\sqrt{\mathrm{Hz}}$, which was an average value between 0.01 Hz to 1 Hz. The approximated ARW was consistent with the Allan Variance in Figure 16b. To calculate the Allan Variance, the gyroscope output rate was averaged to 1 Hz, and the data was recorded for 5400 s.

**Figure 17:**
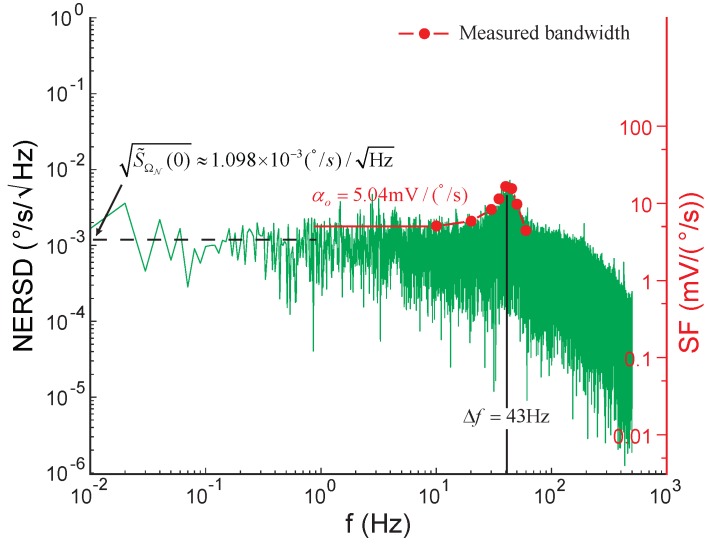
The NER spectral density of the open-loop gyroscope tuned by a DC voltage of 5 V and the corresponding gyroscope frequency response.

Next, a DC voltage of 5 V was applied to the frequency tuning electrodes, resulting in a decrease in Δf from 66 Hz to 43 Hz. Figure 17 demonstrates the NER spectral density of the open-loop gyroscope and the measured gyroscope frequency response. By comparing Figure 16a and Figure 17, it can be found that the open-loop scale-factor increased by 1.546 times as the frequency split decreased by a factor of 1.535, in agreement with Equation (25). However, the ARW only improved by 1.19 times, suggesting that for the gyroscope under test, the ARW was significantly affected by both the mechanical noise and the electrical noise.

**Figure 18:**
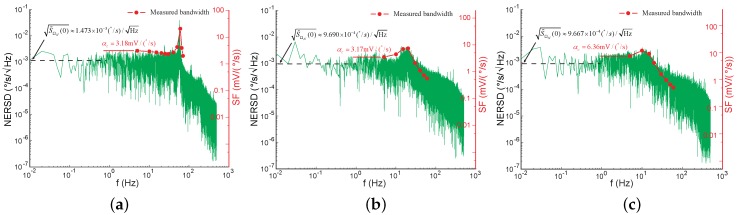
The NER spectral density of the gyroscope in force-rebalance operation. (**a**) In the mode-split case. (Δf=66Hz); (**b**) In the mode-matched case (The tuning voltage was 8.2 V). (**c**) In the mode-matched case with a half $K_{f}$.

Finally, Figure 18 presents the NER spectral density of the gyroscopes in the force-rebalance operation. Revealed from Figure 16a and Figure 18a, the loop closing of the sense channel did not improve the ARW, as predicted in Table 2. Figure 18a,b manifest that the mode matching did not change the scale-factor in the force-rebalance operation but improved the ARW by 1.52 times. The unremarkable improvement of the ARW was due to the considerable mechanical noise. Figure 18c was obtained by reducing $V_{D}$ in Equation (6) from 5 V to 2.5 V, resulting in a half $K_{f}$. The corresponding scale-factor was increased by a factor of 2, which was in agreement with Equation (14). The ARW was not significantly affected by the change of $K_{f}$. From the discussion in Section 5, $K_{f}$ would not influence the ARW attributable to the mechanical noise or the pickoff electrical noise. If the impact of the feedback electrical noise is negligible, the design of the feedback forcer is irrelevant to the noise performance. 

In summary, in both the open-loop operation and the force-rebalance operation, the decrease of the modal frequency split improved the ARW due to the decrease of the electrical noise, as presented in Table 2. However, the mechanical noise was not affected by the modal frequency split, resulting in a moderate improvement of the ARW through mode matching. To further improve the ARW of the gyroscopes under the mode-matched situations, the boost of quality factor is essential. The closing of the sense loop did not significantly change the ARW according to the experimental data, showing an agreement with the models demonstrated in previous sections. The gain of the feedback forcer did not distinctly affect the overall ARW of the gyroscopes, as discussed in Section 5. This result can give very important guidance on the structural design of the feedback forcers, implying that the gain of the forcers is irrelevant to the noise consideration.

## 7. Conclusions

In this paper, we have presented a theoretical analysis of mechanical and electrical noise in the sense channel of MEMS vibratory gyroscopes. The closed-form expressions for the PSD of the NER of gyroscopes in the open-loop and the force-rebalance operations have been obtained. In the open-loop noise model, the features of the noise after the demodulation are investigated by the averaged PSD model. In the force-rebalance noise model, the noise sources are separated from the closed-loop, and the modulation–demodulation process in the closed-loop is linearized by the equivalent transfer function. Via this approach, the closed-loop noise model can be equivalent to an open-loop one and, hence, the analysis method used in the open-loop model can be applied. The analytic models are in close agreement with the numerical models, verifying the validity of the closed-form expressions. The influences of the structure parameters, such as modal frequency split, quality factor, and the gain of the feedback forcer, as well as the gain of the signal conditioning circuit, on the gyroscope noise features have been theoretically analyzed by using the PSD model. In addition, the ARW and the standard deviation of the NER also have been discussed. The impacts of the modal frequency split, the loop closing, the mode matching, and the gain of the feedback forcer on the gyroscope noise characteristics were verified through a MEMS vibratory gyroscope with a tunable modal frequency split.

In both the open-loop and the force-rebalance operations, the decrease of the modal frequency split improved the ARW due to the decrease of the electrical noise. However, the mechanical noise was not affected by the modal frequency split. To further improve the ARW of the gyroscopes under the mode-matched situations, it is important to boost the quality factor. The loop closing did not significantly change the ARW according to the experimental data, which is in agreement with the models proposed in this paper. The gain of the feedback forcer will not distinctly affect the ARW as long as the electrical noise at the feedback node is negligible.

## Figures and Tables

**Table 1:** Default values of the parameters used in numerical simulations.

Parameters	Values	Parameters	Values
*m*	$6\times 10^{-7}\;\mathrm{kg}$	η	1
$f_{d}$	4000Hz	Δf	500Hz
$Q_{y}$	200	$A_{x}$	10μm
$K_{xv}$	$5\times 10^{5}\;V/m$	$K_{a}$	20V/V
$K_{f}$	$5\times 10^{-7}\;N/V$	$f_{l0}$	50Hz
$K_{p}$	0	$K_{i}$	500
$S_{Nm}\left(f\right)$	$7\times 10^{-25}\;N^{2}/\mathrm{Hz}$	$S_{Nep}\left(f\right)$	$(1\times 10^{-13}+2\times 10^{-12}/|f|)\;V^{2}/\mathrm{Hz}$
$S_{Nef}\left(f\right)$	$(1\times 10^{-15}+2\times 10^{-14}/|f|)\;V^{2}/\mathrm{Hz}$		

**Table 2:** Comparison of ${\tilde{S}}_{\Omega_{N}}\left(0\right)$ between different operations of vibratory gyroscopes.

${\tilde{S}}_{\Omega_{N}}\left(0\right)$	Attributable to $N_{m}$	Attributable to $N_{\mathrm{ep}}$	Attributable to $N_{\mathrm{ef}}$
Open-loop, mode-split	$k_{B}T/\eta^{2}A_{x}^{2}m\omega_{y}Q_{y}$	$4k_{B}TR_{p}\Delta \omega^{2}/\eta^{2}A_{x}^{2}K_{xv}^{2}$	−
Open-loop, mode-matched	$k_{B}T/\eta^{2}A_{x}^{2}m\omega_{y}Q_{y}$	$k_{B}TR_{p}\omega_{y}^{2}/\eta^{2}A_{x}^{2}K_{xv}^{2}Q_{y}^{2}$	−
Force-rebalance, mode-split	$k_{B}T/\eta^{2}A_{x}^{2}m\omega_{y}Q_{y}$	$4k_{B}TR_{p}\Delta \omega^{2}/\eta^{2}A_{x}^{2}K_{xv}^{2}$	$k_{B}TR_{f}K_{f}^{2}/\eta^{2}A_{x}^{2}m^{2}\omega_{y}^{2}$
Force-rebalance, mode-matched	$k_{B}T/\eta^{2}A_{x}^{2}m\omega_{y}Q_{y}$	$k_{B}TR_{p}\omega_{y}^{2}/\eta^{2}A_{x}^{2}K_{xv}^{2}Q_{y}^{2}$	$k_{B}TR_{f}K_{f}^{2}/\eta^{2}A_{x}^{2}m^{2}\omega_{y}^{2}$

## Abbreviations

The following abbreviations are used in this manuscript:

ACF	autocorrelation function
AGC	automatic gain control
ARW	angle random walk
DC	direct current
FPGA	field-programmable gate array
LPF	low pass filter
MEMS	micro-electro-mechanical systems
NER	noise equivalent rate
PLL	phase-locked loop
PSD	power spectral density

## Nomenclature

$A_{x}$	vibration amplitude of the x-axis, m	*B*	frequency band of interest, Hz
c(t)	inverse Laplace transform of C(s)	$c_{y}$	damping coefficient of the sense axis, N/(m/s)
$C_{fb}$	capacitance of the feedback electrodes, F	C(s)	transfer function of the PI controller and the LPF
$f_{d}$	vibration frequency of the x-axis, Hz	$f_{l0}$	cutoff frequency of the LPF, Hz
$f_{res}$	frequency resolution in estimation of $\sigma_{\Omega_{N}}$, Hz	$f_{x}$	natural frequency of the x-axis, Hz
$f_{y}$	natural frequency of the y-axis, Hz	$F_{c}$	Coriolis force, N
$F_{x}$	force exerted along the x-axis, N	$F_{y}$	force exerted along the y-axis, N
$g_{y}\left(t\right)$	inverse Laplace transform of $G_{y}\left(s\right)$	$G_{y}\left(s\right)$	transfer function of the sense axis
H(s)	equivalent transfer function of modulated–demodulated $G_{y}\left(s\right)$	*j*	imaginary unit, $\sqrt{-1}$
$k_{1}$	a constant for the flicker noise at the pickoff circuit, V${}^{2}$	$k_{2}$	a constant for the flicker noise at the feedback circuit, V${}^{2}$
$k_{B}$	Boltzmann constant, $1.38\times 10^{-23}$ J/K	$k_{x}$	stiffness of the x-axis, N/m
$k_{y}$	stiffness of the y-axis, N/m	$K_{a}$	gain of the signal conditioning circuit, V/V
$K_{DA}$	gain of the digital-to-analog converter buffer, V/V	$K_{f}$	gain of the feedback forcer, N/V
$K_{i}$	integral parameter of the PI controller	$K_{p}$	proportional parameter of the PI controller
$K_{xv}$	gain of the pickoff circuit (displacement to voltage), V/m	L(s)	transfer function of the LPF
*m*	mass of the sensor resonator, kg	N(t)	noise in the sense channel before modulation, V
$N_{c}\left(t\right)$	noise in the sense channel after modulation, V	$N_{ef}\left(t\right)$	electrical noise introduced by feedback circuits, V
$N_{ep}\left(t\right)$	electrical noise introduced by pickoff circuits, V	$N_{m}\left(t\right)$	mechanical-thermal noise, N
$Q_{y}$	quality factor of the y-axis	$R_{f}$	equivalent resistance of the Johnson noise at the feedback circuit, Ω
$R_{p}$	equivalent resistance of the Johnson noise at the pickoff circuit, Ω	$R_{N}\left(\tau \right)$	ACF of N(t)
$R_{N_{c}}\left(\tau \right)$	ACF of $N_{c}\left(t\right)$	$S_{N}\left(f\right)$	PSD of N(t), V${}^{2}$/Hz
$S_{N_{c}}\left(f\right)$	PSD of $N_{c}\left(t\right)$, V${}^{2}$/Hz	$S_{Nef}\left(f\right)$	PSD of $N_{ef}\left(t\right)$, V${}^{2}$/Hz
$S_{Nep}\left(f\right)$	PSD of $N_{ep}\left(t\right)$, V${}^{2}$/Hz	$S_{Nm}\left(f\right)$	PSD of $N_{m}\left(t\right)$, N${}^{2}$/Hz
$S_{\Omega_{N}}\left(f\right)$	PSD of the NER, (rad/s)${}^{2}$/Hz	*T*	temperature, K
$T_{a}$	time for averaging, s	$T_{\Omega }\left(s\right)$	equivalent transfer function in force-rebalance sense channel
$T_{N}\left(s\right)$	equivalent transfer function in force-rebalance noise model	$V_{D}$	bias DC voltage applied to the forcer electrodes, V
*x*	displacement along the x-axis, m	*y*	displacement along the y-axis, m
$\alpha_{o}$	open-loop scale-factor, V/(rad/s)	$\alpha_{c}$	closed-loop scale-factor, V/(rad/s)
Δf	modal frequency split, Hz	Δω	modal frequency split, rad/s
η	angular gain factor	$\sigma_{\Omega }$	standard deviation of the gyroscope output, rad/s
$\sigma_{\Omega_{N}}$	standard deviation of NER, rad/s	$\varphi_{d}$	demodulation phase, rad
$\varphi_{od}$	optimal demodulation phase, rad	$\omega_{d}$	vibration frequency of the x-axis, rad/s
$\omega_{x}$	natural frequency of the x-axis, rad/s	$\omega_{y}$	natural frequency of the y-axis, rad/s
Ω(s)	Laplace transform of Ω(t)	$\hat{\Omega }\left(s\right)$	Laplace transform of $\hat{\Omega }\left(t\right)$
Ω(t)	input angular rate, rad/s	$\hat{\Omega }\left(t\right)$	measured angular rate, rad/s
$\Omega_{ARW}$	angle random walk, (rad/s)/$\sqrt{\mathrm{Hz}}$		
